# Psoriasis Vulgaris Masked by Tinea Pedis in Two Unhoused Patients

**DOI:** 10.7759/cureus.65206

**Published:** 2024-07-23

**Authors:** Shandelle Sookdar, David F Pupaza, Paul A Alvarez, Linda Washington-Brown, Peter Gutierrez, Damian Casadesus

**Affiliations:** 1 Internal Medicine, St. George's University School of Medicine, Miami, USA; 2 Internal Medicine, Hospital Corporation of America (HCA) Westside/Northwest, Davie, USA; 3 Internal Medicine, St. George's University School of Medicine, Pembroke Pines, USA; 4 Family Medicine, Miami Rescue Mission Clinic, Hollywood, USA; 5 Internal Medicine, Jackson Memorial Hospital, Miami, USA

**Keywords:** atypical rash, confirmation bias, tinea pedis, cellulitis, psoriasis

## Abstract

Psoriasis is a chronic inflammatory disease characterized by clearly marginated silvery plaques that affect men and women equally. Symptoms can vary among individuals; typically, it presents on the scalp, elbows, and knees. We present two cases of patients initially diagnosed with tinea pedis who showed no improvement with medical treatment. The first patient is an African American male in his 50s who arrived at a free clinic for unhoused persons with lesions to both feet initially diagnosed as tinea pedis. Although the patient was compliant with applying topical formulations of tolnaftate and clotrimazole, there was no discernible improvement in his symptoms and the skin lesions. After a thorough examination of the skin throughout the entire body, the diagnosis of psoriasis was considered. The patient started treatment with steroidal cream with improvement of the symptoms and lesions. The second patient is a Caucasian male in his 20s who also presented initially with complaints of a dry, intensely pruritic, and scaly rash on the dorsum of both his feet, as well as in between the digits of his feet for which an initial diagnosis of tinea pedis was also made. The patient remained non-compliant with treatment and, after reevaluation of his lesions along with an extensive survey of his body, was deemed to have psoriasis and prescribed topical hydrocortisone. The patient continued to remain non-compliant with his therapeutic regimen and subsequently developed cellulitis which is yet to resolve with treatment.

## Introduction

Plaque psoriasis, a chronic inflammatory disease caused by genetic predisposition and autoimmune pathogenicity, affects the skin. The incidence of psoriasis in the USA is 2%-3%, representing approximately 7.5 million adults aged 20 years or older [1,2], with most cases affecting Caucasian Americans. Psoriasis is less prevalent among African American and Asian populations [3,4]. There is limited epidemiologic research on plaque psoriasis in the non-Caucasian population, with an increased occurrence of undiagnosed psoriasis in these populations.

Classic features of psoriatic skin manifestations are raised erythematous plaques with silvery-white scales that commonly appear on the scalp, torso, and limbs. Psoriasis involves cycles of flares lasting weeks to months, followed by remission. Palmoplantar psoriasis is an uncommon form of psoriasis, and it can be difficult to differentiate from other medical conditions affecting the feet [5]. We present a patient diagnosed and treated for tinea pedis, and then another patient who developed cellulitis as a complication of untreated psoriasis [6].

## Case presentation

Case 1

An African American male in his 50s, a resident of a shelter house, who had a past medical history of diabetes mellitus, ulcerative colitis, and hypertension, presented to the outpatient clinic with a two-week history of a progressive pruritic rash to the dorsum of both feet. The rash was located between the first and second toes of each foot and associated with itchiness. The patient’s past medical history revealed that he was seen and treated at a local hospital about four months prior for similar symptoms along with drainage of pus from the dorsum of both feet. He was discharged on cephalexin 500 mg twice daily for 7 days. Following the completion of the oral antibiotic treatment, the patient was treated with 1% topical tolnaftate cream for one week followed by 3 weeks of 1% topical Clotrimazole for tinea pedis.

**Figure 1:**
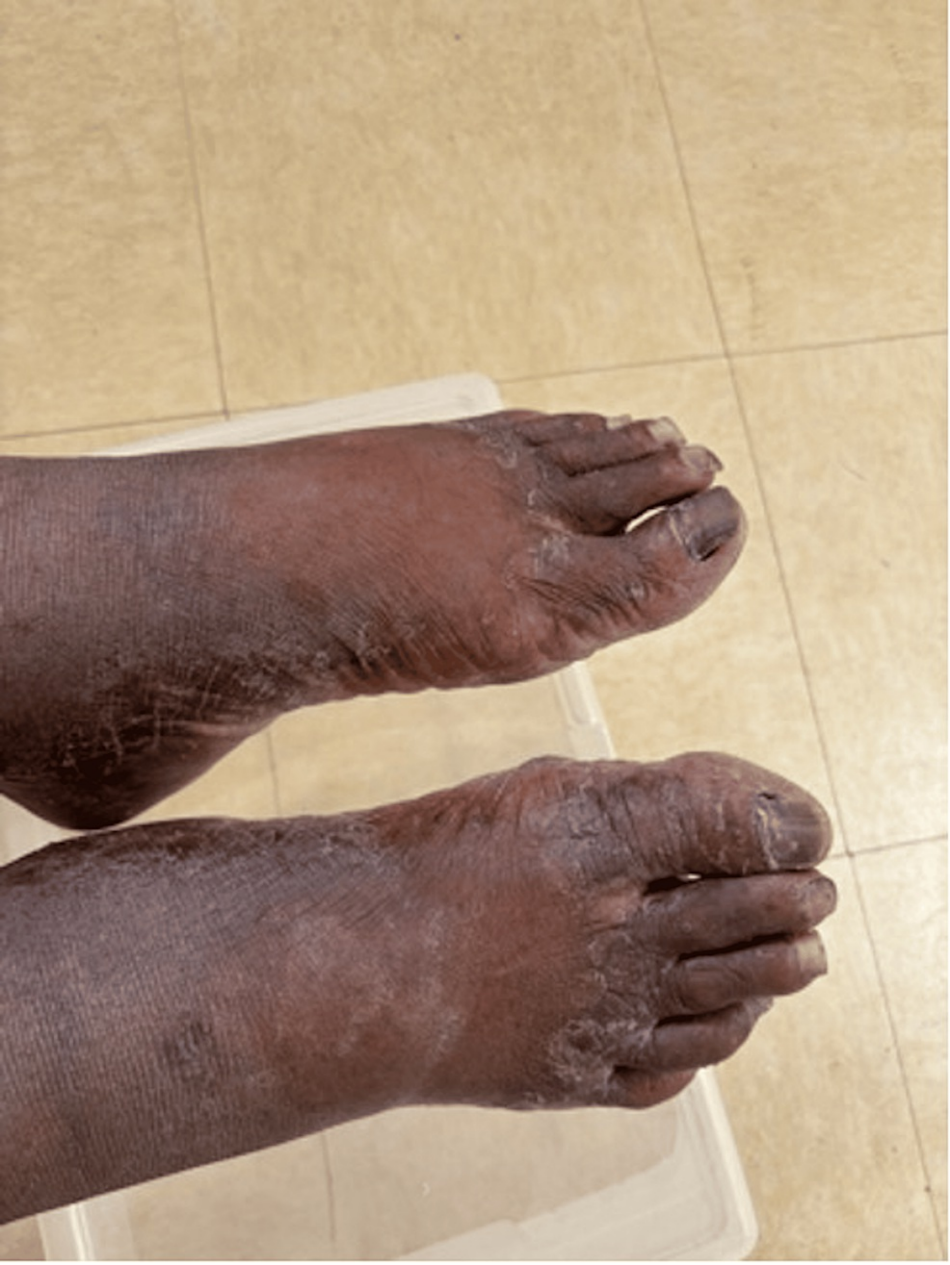
Draining malodorous lesion between the first and second digits, and dry, cracked areas between the second, third, and fourth digits of both feet.

A review of systems revealed reoccurring complaints of rashes on both feet since his admission to the residential facility. The rashes were silver and appeared plaque-like at both elbows, posterior knees, and bilateral dorsal surfaces of the feet. Foul-smelling lesions draining cloudy fluid were reported between the first and second digits of his feet along with dry and broken areas of skin between the second, third, and fourth digits of both feet. The patient had been experiencing such pruritic lesions on both elbows, as well as portions of the proximal radial and ulnar regions, and the posterior knee areas for several years. During this presentation, the vital signs were within normal limits. On examination, cardiopulmonary and abdominal examinations were normal. The skin examination revealed a draining malodorous lesion between the first and second digits, and dry, cracked areas between the second, third, and fourth digits of both feet (Figure 1).

**Figure 2:**
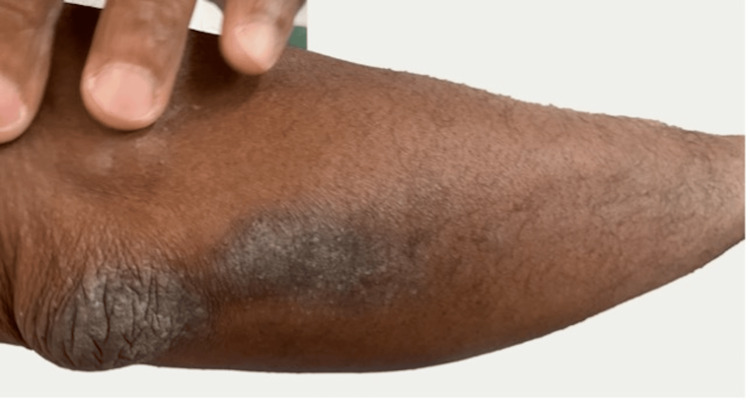
Silvery hyperpigmented plaque formation to both elbows.

**Figure 3:**
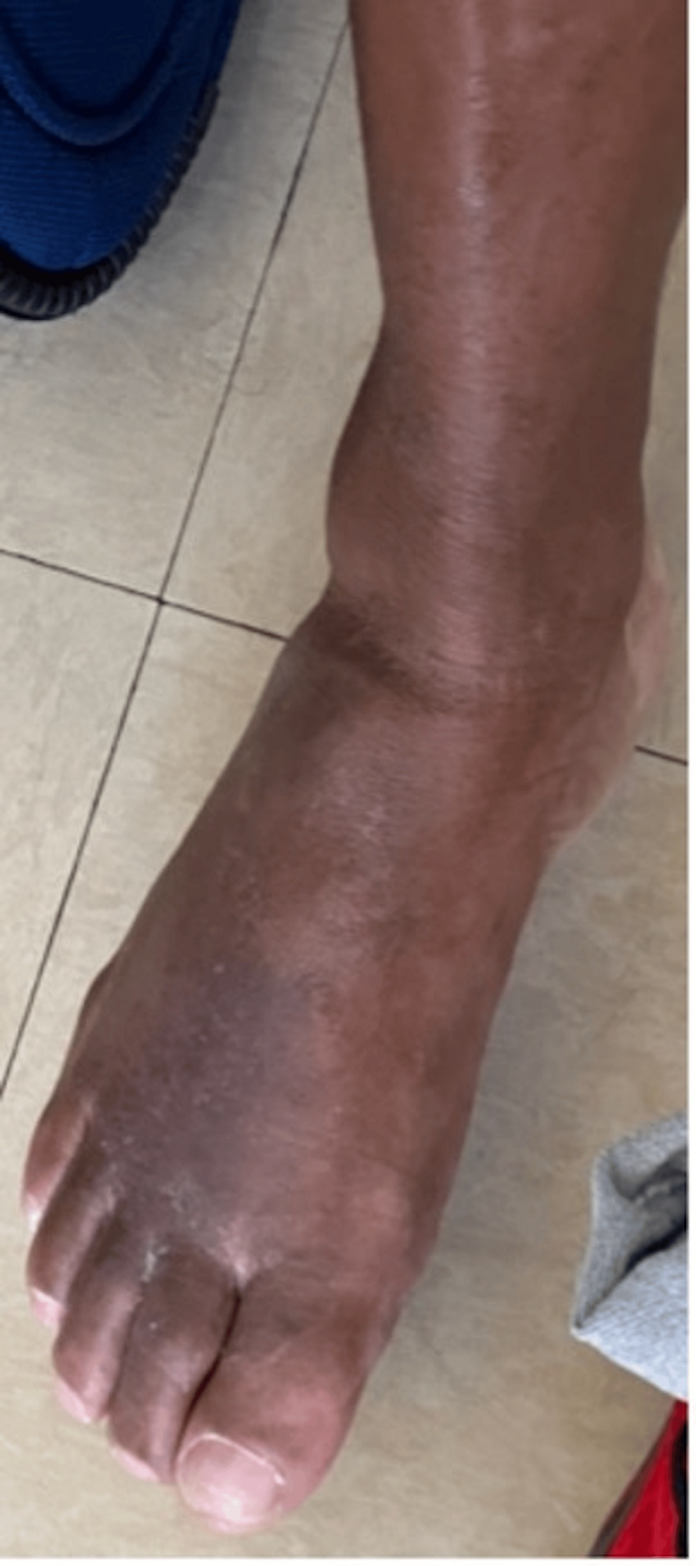
Normal skin after completed treatment with steroidal cream.

The patient confirmed that he was adherent to medical treatment, however, he had minimal relief from his symptoms. He was advised to continue medical management with the antifungal medication and return to the office for a follow-up in one week. Two weeks later, the patient continued with itchiness and poor wound healing. On a more thorough examination, both feet showed lichenification, and a silvery hyperpigmented plaque formation to both elbows, posterior knee, and dorsal surfaces of the feet (Figure 2). The diagnosis of plaque psoriasis was established, and the patient was prescribed hydrocortisone 2.5% twice a day to all affected areas on the dorsum of both feet, elbows, posterior knees, and posterior aspects of both lower arms where plaques were observed. Follow-up one week later revealed improvement in symptoms and skin lesions (Figure 3).

Case 2

**Figure 4:**
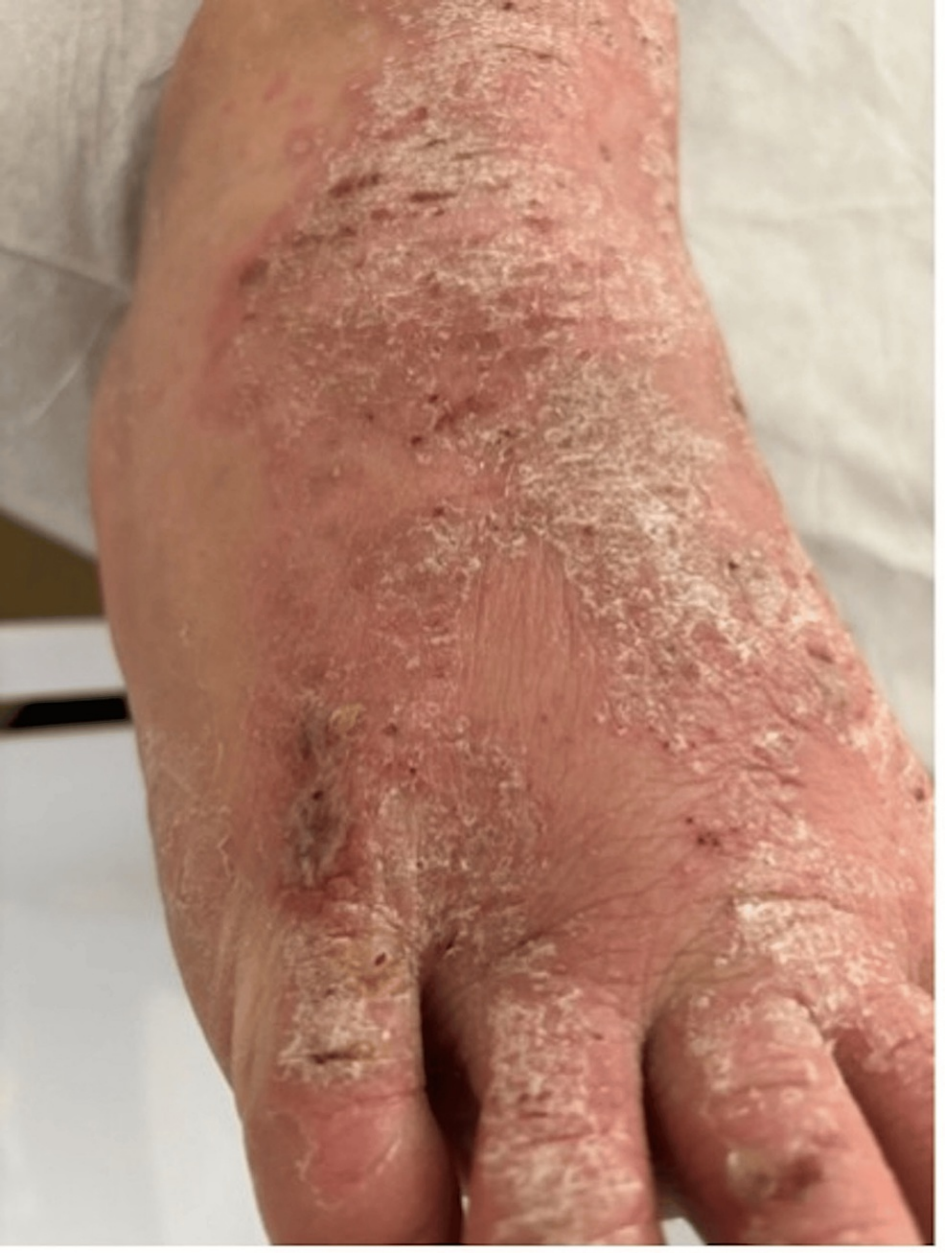
Silvery plaques that were actively bleeding and intensely pruritic were observed on the dorsum of both feet.

**Figure 5:**
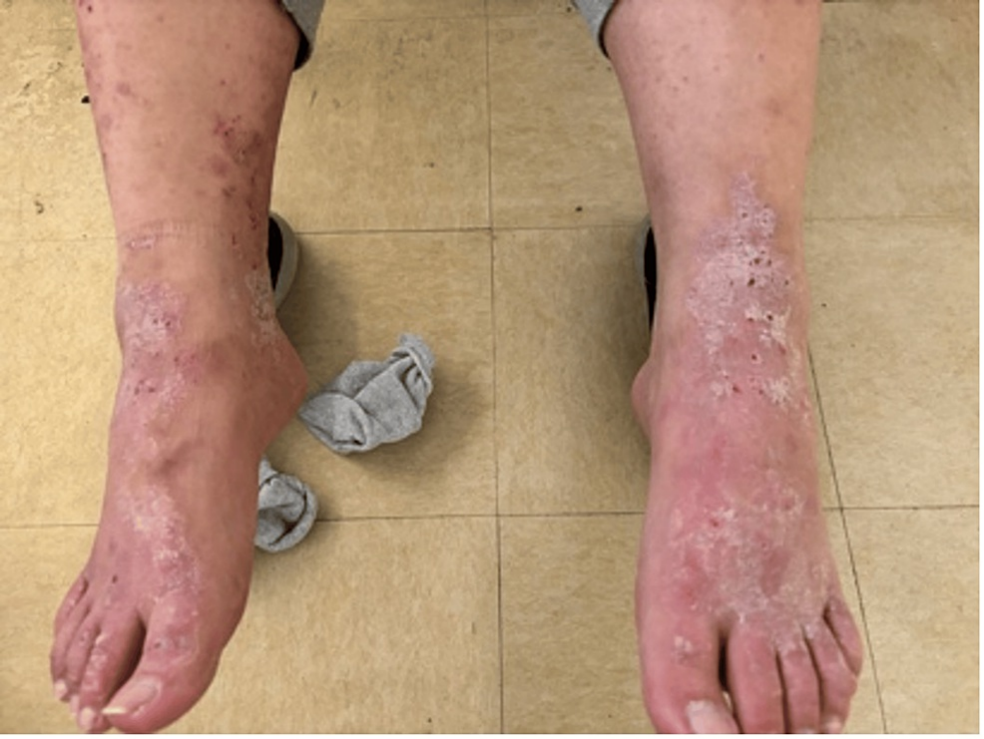
Improvement of the skin lesions after treatment.

A Caucasian male in his 20s with a past medical history of Crohn’s disease with a colostomy bag insertion and multiple abdominal hernia repairs presented to the outpatient clinic with a rash on each of his feet. The patient was a resident of the same homeless shelter as the patient above and visited the same outpatient clinic. The patient described two months of flaky, intensely pruritic erythematous rashes between the digits and on the dorsum of both feet. Vital signs were within normal limits and abdominal and cardiopulmonary examinations were unremarkable. Upon further examination, a plaque-like rash was observed between the digits and on the dorsum of both feet. He was diagnosed with tinea pedis because of a supposed uptick in cases seen within the same homeless shelter at the time of the evaluation. He was subsequently prescribed clotrimazole but was not compliant with the treatment and returned to the clinic two weeks later with a severe exacerbation of his symptoms. Further examinations revealed several crusted, silvery plaques on an erythematous base which were intensely pruritic and accompanied by punctate hemorrhages in the previously affected areas. A more general skin survey demonstrated similar lesions at the extensor surface of both elbows, which the patient said had also been causing him discomfort but forgot to mention at the original encounter. All of the lesions were flaking, desquamated, and draining a clear serosanguinous fluid. He started hydrocortisone cream 1%, but he remained non-compliant and developed bilateral foot cellulitis with severe pain, ulcerations, and serosanguineous discharge associated with fever, nausea, and vomiting (Figure 4). The patient’s care was entrusted to a local hospital, where he was treated with Keflex 500 mg orally for 7 days, CeraVe 46.5% ointment for 2 weeks, mupirocin 2 % for three days, and tramadol 50 mg for three days as needed. At the time of writing the manuscript, the patient’s symptoms demonstrated some improvement with the erythematous, scaly, and pruritic lesions still present over the psoriasis scabs (Figure 5).

## Discussion

Both tinea pedis and psoriasis can be chronic and cause skin scaling and redness, itching, and involvement of the nails [7]. Essential aspects of the physical examination that allow the clinician to distinguish psoriasis from tinea pedis is that tinea pedis primarily affects the skin on the feet. In contrast, psoriasis can affect various body parts, including the feet. Both can involve the skin and the toenails and may recur [8]. Accurate differentiation between tinea pedis and psoriasis is paramount in clinical practice due to the potential for exacerbation with inappropriate treatments. For instance, terbinafine, commonly used to treat fungal infections like tinea pedis, can exacerbate psoriasis if misdiagnosed. Conversely, corticosteroids, effective in managing psoriasis, can worsen fungal infections like tinea pedis if applied incorrectly. This distinction underscores the necessity of thorough evaluation and diagnostic precision to tailor appropriate therapies effectively.

A low incidence of psoriasis is reported in people of color due to the reduced availability of research in these groups and difficulty identifying lesions [3,4]. This low incidence is partly due to the different appearance of psoriasis on black or brown skin compared to white skin, making diagnosis more challenging. Additionally, this underdiagnosis can result from a reduced awareness of psoriasis among this population, leading to underreporting of symptoms and delayed seeking of medical attention. In the homeless population, as in our patient, psoriasis is more underdiagnosed, and tinea pedis is probably more commonly noted [9]. Financial limitations can lead to reduced access to healthcare services due to financial constraints, resulting in delays in diagnosis, insufficient treatment, and an inability to afford necessary medications for the treatment of psoriasis [10]. Furthermore, the scarcity of clean water, bathing facilities, and laundry services among the unhoused limits their ability to effectively manage these medical conditions. Exposure to adverse outdoor conditions, including cold, dry air, or excessive heat, is more prevalent among the unhoused, irritating the skin and triggering flare-ups. Moreover, poor nutrition, smoking, excessive alcohol consumption, and limited opportunities for regular exercise are more prevalent in unhoused populations, thereby contributing to a heightened prevalence and severity of psoriasis.

## Conclusions

Psoriasis is associated with an indolent course and presents as silvery plaques on the scalp, elbows, and knees. It can also affect the feet similarly to tinea pedis, causing redness and itchiness. A rigorous and thorough physical examination that prioritizes objectivity and inclusion is a key part of the physician’s ability to rule in or out similar medical conditions. Eventually, as seen in patients like ours, this can prove to be crucial in treating debilitating conditions like psoriasis, for which rare complications like cellulitis can be severe. Oftentimes as physicians, preconceived notions related to demographic groups or past experiences can influence what a provider chooses to look for when examining a patient. Cases such as these shed light on the socio-economic barriers that contribute to underdiagnosis and inadequate management of psoriasis among vulnerable populations. Moving forward, raising awareness, improving access to healthcare, and enhancing dermatologic education are pivotal in improving outcomes and quality of life for such patients.
